# The Abbe Flap for Upper Lip Reconstruction

**Published:** 2014-08-27

**Authors:** Theodore T. Nyame, Abraham Pathak, Simon G. Talbot

**Affiliations:** Division of Plastic Surgery, Department of Surgery, Brigham and Women's Hospital, Boston, Mass

**Keywords:** lip reconstruction, Abbe flap, lip defects, labial blood supply, oral competence

## DESCRIPTION

A 37-year-old otherwise healthy man presents after a dog bite with avulsion of approximately 50% of the upper lip and loss of the amputated part.

## QUESTIONS

1. **Discuss the key principles of lip reconstruction.**
2. **Describe the history and classical description of the Abbe flap.**
3. **What indications and injuries are amenable to an Abbe flap?**
4. **What are the key anatomical features of the lips?**

## DISCUSSION

The goals of lip reconstruction are both functional and aesthetic. Oral competence, muscle integrity, and adequate stomal aperture are critical to a functional lip reconstruction. Respect for the anatomic landmarks of the lip, such as the white roll or vermilion-cutaneous junction, allow for a cosmetically natural reconstruction. The unique structure and appearance of lips lends itself to using existing lip tissue for “like with like” reconstruction whenever possible, as distant tissue typically yields inferior results. This notion further allows preservation of a contiguous, innervated orbicularis oris muscle to render a better functional reconstruction. Local flaps including cross-lip flaps have become mainstays of reconstruction for larger deformities not amenable to direct or sliding lip closure.

The first reported case of a 2-stage pedicled “lip switch” flap is credited to Sabattini in 1838. However, the use of a flap based on the labial branches of the facial artery was popularized by Dr Robert Abbe in 1898 as a complete philtral reconstruction for bilateral cleft lip deformities.^1^ As initially described, the Abbe flap is designed with a width approximately half that of the defect (to adequately share the transverse length discrepancy between the existing lips) with the height of the flap equaling the vertical dimension of the defect. The flap is designed with a pedicle toward the side of the defect. Where possible, the central lower lip is used as a donor site, as this is typically hair-bearing in males and leaves the least visible scar. The white roll should be marked ahead of time as it may become obscured by pallor or bleeding. The flap is elevated including skin, muscle, and mucosa with care at the vermilion border to preserve a small amount of mucosa and the labial artery. The flap is rotated and inset, taking care to align anatomic landmarks including closure of the orbicularis oris and matching of the white roll. Careful eversion of sutured edges prevents “notching” and minimizes scarring. After 2 to 3 weeks, the pedicle is divided and the flap inset.^1^^,^^3^

These full-thickness flaps are now referred to as Abbe flaps, with many described modifications having firmly established their role in reconstruction of both upper and lower lip defects due to congenital anomalies, trauma, or neoplasia.^2^ The principal indication for the Abbe flap is a full-thickness defect involving one-third to two-thirds of the lip with an intact oral commissure.^3^^,^^4^ It is important to note that in the acute setting the available tissue should be used as best as possible to achieve wound closure. Following closure, a period of wound contracture is allowed to minimize the defect size. The Abbe flap should not be performed in the ED or in an acute dog bite situation. Reconstruction prior to this period of wound contracture would require a large amount of donor tissue, have a high risk of infectious complications, and potentially lead to poorer results.

A solid understanding of cutaneous anatomy is important in flap design and elevation. Depending on the defect location, the Abbe flap pedicle is the superior or inferior labial artery, originating from the facial artery just lateral to the oral commissure. These vessels then course in a horizontal plane deep to mucosa overlying the orbicularis oris muscle to anastomose at the midline with contralateral branches. The superior labial artery provides blood supply to the upper lip with terminal branches supplying the nasal alae and septum. The inferior labial artery provides blood supply to the lower lip and superior part of the chin.^5^ Sensation to the upper lip is provided via the infraorbital nerves, and to the lower lip by the mental nerves emerging through the mental foramina. Small cutaneous branches are cut during flap elevation but spontaneous sensory recovery is often excellent.

The Abbe flap is an excellent choice for reconstruction of full-thickness defects of the lip excluding the oral commissure. For more than 100 years, it has remained a versatile technique for upper and lower lip reconstruction, providing good functional and aesthetic results. Its use is sure to persist as an example of “like with like” reconstruction.

## Figures and Tables

**Figure 1:**
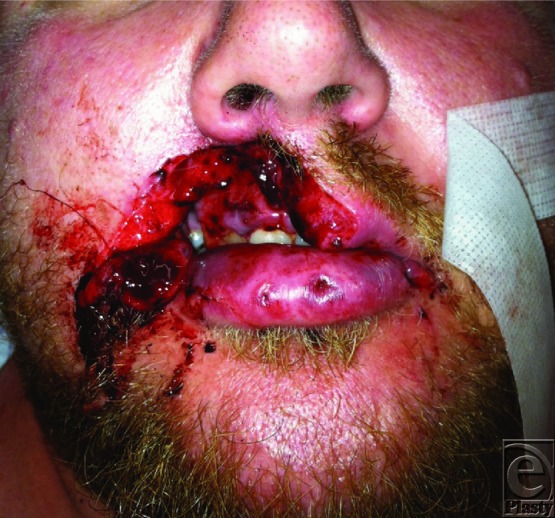
Dog bite defect comprising approximately half of the right upper lip.

**Figure 2:**
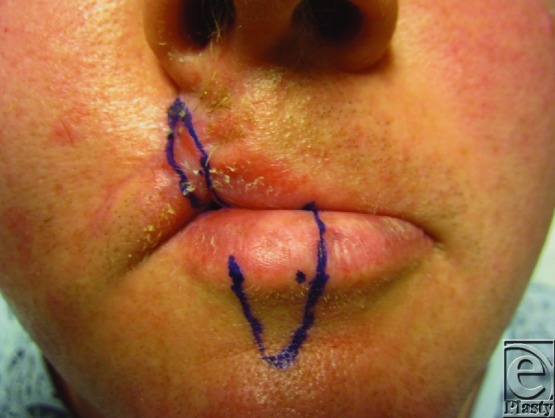
Abbe flap design utilizing the central portion of the lower lip with the pedicle toward the anticipated defect.

**Figure 3:**
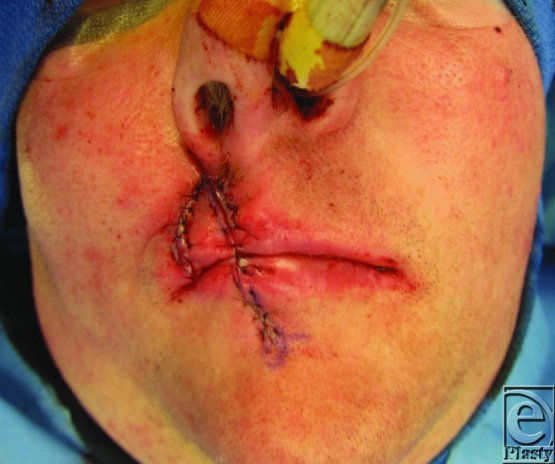
Flap elevation and initial inset.

**Figure 4:**
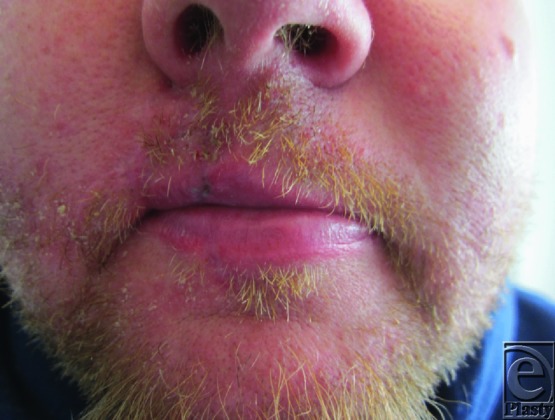
Final appearance after pedicle division, inset, and healing.
